# Distinct Expression Levels and Patterns of Stem Cell Marker, Aldehyde Dehydrogenase Isoform 1 (ALDH1), in Human Epithelial Cancers

**DOI:** 10.1371/journal.pone.0010277

**Published:** 2010-04-21

**Authors:** Shan Deng, Xiaojun Yang, Heini Lassus, Shun Liang, Sippy Kaur, Qunrui Ye, Chunsheng Li, Li-Ping Wang, Katherine F. Roby, Sandra Orsulic, Denise C. Connolly, Youcheng Zhang, Kathleen Montone, Ralf Bützow, George Coukos, Lin Zhang

**Affiliations:** 1 Center for Research on Early Detection and Cure of Ovarian Cancer, University of Pennsylvania, Philadelphia, Pennsylvania, United States of America; 2 Department of Obstetrics and Gynecology, University of Pennsylvania, Philadelphia, Pennsylvania, United States of America; 3 Abramson Family Cancer Research Institute, University of Pennsylvania, Philadelphia, Pennsylvania, United States of America; 4 Department of Pathology and Laboratory Medicine, University of Pennsylvania, Philadelphia, Pennsylvania, United States of America; 5 Department of Obstetrics and Gynecology, Peking Union Medical College, Chinese Academy of Medical Sciences, Beijing, People's Republic of China; 6 Department of General Surgery, Lanzhou University Second Hospital, Lanzhou, People's Republic of China; 7 Department of Obstetrics & Gynecology and Pathology, University of Helsinki and Helsinki University Central Hospital, Helsinki, Finland; 8 Center for Reproductive Sciences, University of Kansas, Kansas City, Kansas, United States of America; 9 Women's Cancer Research Institute, Cedars-Sinai Medical Center, Los Angeles, California, United States of America; 10 Women's Cancer Program, Fox Chase Cancer Center, Philadelphia, Pennsylvania, United States of America; Karolinska Institutet, Sweden

## Abstract

Aldehyde dehydrogenase isoform 1 (ALDH1) has been proved useful for the identification of cancer stem cells. However, our knowledge of the expression and activity of ALDH1 in common epithelial cancers and their corresponding normal tissues is still largely absent. Therefore, we characterized ALDH1 expression in 24 types of normal tissues and a large collection of epithelial tumor specimens (six cancer types, n = 792) by immunohistochemical staining. Using the ALDEFUOR assay, ALDH1 activity was also examined in 16 primary tumor specimens and 43 established epithelial cancer cell lines. In addition, an ovarian cancer transgenic mouse model and 7 murine ovarian cancer cell lines were analyzed. We found that the expression levels and patterns of ALDH1 in epithelial cancers are remarkably distinct, and they correlate with their corresponding normal tissues. ALDH1 protein expression levels are positively correlated with ALDH1 enzymatic activity measured by ALDEFLUOR assay. Long-term *in vitro* culture doesn't significantly affect ALDH1 activity in epithelial tumor cells. Consistent with research on other cancers, we found that high ALDH1 expression is significantly associated with poor clinical outcomes in serous ovarian cancer patients (n = 439, p = 0.0036). Finally, ALDH^br^ tumor cells exhibit cancer stem cell properties and are resistant to chemotherapy. As a novel cancer stem cell marker, ALDH1 can be used for tumors whose corresponding normal tissues express ALDH1 in relatively restricted or limited levels such as breast, lung, ovarian or colon cancer.

## Introduction

Research has provided strong support for the cancer stem cell hypothesis, which proposes that a relatively rare subpopulation of tumor cells have the unique ability to initiate and perpetuate tumor growth [1], [2], [3], [4], [5], [6], [7], [8], [9], [10]. These cells, called cancer stem cells or tumor-initiating cells, share various characteristics with embryonic and somatic stem cells including self-renewal and multi-potent differentiation [11], [12], [13], [14], [15], [16], [17]. Cancer stem cells may be highly resistant to radiation or chemotherapy [18], [19], [20], therefore, the development of more effective therapies for cancer requires effective targeting of this cell population [11], [12], [13], [14], [15], [16], [17]. Cancer stem cells can be identified and isolated using markers specific for normal progenitor or stem cells of the same organ [16], [21]. Several markers have proved useful for the isolation of subsets enriched for epithelial cancer stem cells, including CD44/CD24 (HAS/PGP1) [3], CD133 (PROM1) [4], ATP-binding cassette B5 (ABCB5) [22], CD90 (THY1) [23], CD61(β3 Integrin) [24], 26S proteasome activity [25], as well as Hoechst33342 exclusion [6], [26], [27].

Aldehyde dehydrogenase (ALDH) catalyzes the irreversible oxidation of a range of aliphatic and aromatic aldehydes to their corresponding carboxylic acids [28]. High ALDH activity is detected in stem and progenitor cells of various lineages including hematopoietic [29], [30], [31], mesenchymal [32], neural [33], mammary [34], [35] and prostate [36]. The ALDEFLUOR assay was originally developed to detect ALDH activity in hematopoietic tissues; the fluorescent ALDEFLUOR reaction product accumulates in stem cells and correlates with ALDH activity [29]. ALDH converts the ALDH substrate, BAAA (BODIPY-aminoacetaldehyde), into the fluorescent product BAA (BODIPY-aminoacetate), which is retained inside viable cells. Cells expressing high levels of ALDH become brightly fluorescent (ALDH^br^) and can be identified and enumerated using a standard flow cytometer or isolated by cell sorting for further purification and characterization. ALDECOUNT® is an FDA-approved *in vitro* diagnostic product which is based on the ALDEFLUOR assay and is used for the identification and enumeration of stem cells for clinical applications. Most recently, the ALDEFLUOR assay has been successfully applied to detect the progenitor and cancer stem cells in non-hematopoietic tissues such as mammary gland and breast cancers [34]. The ALDEFLUOR reagent is known to act as a substrate for ALDH1. Importantly, an ALDH1 specific antibody can be used to detect cancer stem cells in paraffin-embedded clinical specimens [34]. Several independent groups have reported that ALDH1 expression can be used as a prognostic marker for epithelial cancers [34], [37], [38], [39], [40], [41], [42], [43]. Therefore, the ALDEFLUOR assay and ALDH1 immunostaining may prove useful for the detection and isolation of cancer stem cells in epithelial tumors, thus facilitating the introduction of cancer stem cell concepts to clinical practice [34]. However, our knowledge of the expression and activity of ALDH1 in human epithelial cancers and their corresponding normal tissues, as well as its clinical significance, is still in its infancy. To advance our knowledge in this important area, we characterized ALDH1 expression in 24 types of normal human tissues as well as a large collection of paraffin-embedded human epithelial tumor specimens (six cancer types, n = 792) by immunohistochemical staining. Using the ALDEFUOR assay, ALDH1 activity was also examined in 16 primary tumor specimens and 43 established human epithelial cancer cell lines. In addition, an ovarian cancer transgenic mouse model and 7 murine ovarian cancer cell lines were analyzed in our study.

## Materials and Methods

### Fresh Tumor Specimens

Tissues were obtained after patients' written consent under a general tissue collection protocol approved by the institution's Institutional Review Board (IRB) of the University of Pennsylvania. All specimens were collected at the time of debulking surgery from previously untreated patients with late-stage ovarian cancer. Erythrocytes were lysed with ammonium chloride solution. Single-cell suspensions were prepared using a 40 µm cell strainer. Dead cells were eliminated using magnetic beads (Dead Cell Removal kit, Miltenyi Biotec) and a MidiMACS separator.

### Tissue microarrays

*Tissue array network cohort:* An FDA normal human organ tissue microarray was used to characterize ALDH1 expression in normal organs. This tissue array platform included 24 types of normal human organs based on FDA guidelines, and each organ was sampled from 3 normal individuals. Six types of human epithelial tumor tissue microarrays were used to characterize ALDH1 expression in epithelial tumors. Each patient was represented with at least two core tissue biopsies. *Helsinki cohort:* An ovarian cancer tissue microarray was used to examine the prognostic significance of ALDH1 in ovarian cancer. The array included patients treated for primary serous ovarian carcinoma at the Helsinki University Central Hospital.

### Cell lines and Cell Culture

A total of 50 established cancer cell lines were used in this study including 15 human breast, 18 human ovarian, 7 murine ovarian, and 10 human colon. All cancer cell lines were cultured in RPMI 1640 medium supplemented with 10% fetal bovine serum (Invitrogen). Three independent immortalized human ovarian surface epithelial cells (IOSEs) were generously provided by Dr. Auersperg, and cultured in a 1∶1 combination of medium 199 and MCDB 105 medium (Sigma) supplemented with 15% FBS. Murine ovarian surface epithelial cells (MOSEs) were isolated and cultured as previously reported [44].

### Transgenic Mice

The animal study protocol was reviewed and approved by the institutional animal care and use committee (IACUC) of the University of Pennsylvania. The ovarian cancer MISRII-SV40 transgenic mouse was generated by Dr. Denise Connolly's laboratory [45].

### Immunohistochemistry and Image Analysis

Immunohistochemistry (IHC) was performed using the VECTASTAIN ABC Kit as described by the manufacturer (Vector). The following primary antibodies were used in this study: mouse anti-human ALDH1 (clone: 44/ALDH, 1∶250, BD Pharmingen) [34], [39], [46]; mouse anti-human Ki67 (1∶100, DAKO) and rabbit anti-human CD45 (1∶200, Millipore). Antibodies were incubated overnight at 4°C. The immunoreaction was visualized with 3,3′-diaminobenzidine. Double immunofluorescent staining was performed as previously described [47].

### ALDEFLUOR Assay

ALDH activity was detected using the ALDEFLUOR assay kit (StemCell Technologies) as described by the manufacturer. Briefly, dissociated single cells from cell lines or tumor specimens were suspended in ALDEFLUOR assay buffer containing an ALDH substrate, bodipy-aminoacetaldehyde (BAAA), at 1.5 µM, and incubated for 1 hr at 37°C. A specific inhibitor of ALDH, diethylaminobenzaldehyde (DEAB), at a 10-fold molar excess, was used as negative control. Flow cytometry data was analyzed by BD FACSDiva software V6.1.3 (BD Biosciences) or FlowJo software (TreeStar).

### Protein Isolation and Western blot

Cultured cells were lysed in 200 µl of lysis buffer containing 50 mM Tris-HCl (pH 7.4), 150 mM NaCl, and 1% Triton X-100. Proteins were separated by 10% SDS-PAGE under denaturing conditions and transferred to nitrocellulose membranes. Membranes were incubated with an anti-ALDH1 primary antibody (1∶300, BD Pharmingen), followed by incubation in anti-mouse secondary antibody conjugated with horseradish peroxidase (1∶5,000; Amersham Biosciences). Immunoreactive proteins were visualized using ECL Western Blotting Substrate (Thermo Scientific).

### Mammosphere culture

Mammosphere cultures were performed as described by Dontu *et al* [48]. Briefly, single cells were plated in ultra-low attachment 24-well plates (Corning) at a density of 20,000 viable cells/ml and 5000 cells/ml in subsequent passages. Cells were grown in a serum-free mammosphere culture medium (MammoCult, StemCell Technologies) supplemented with MammoCult Proliferation Supplements. Mammospheres were collected by gentle centrifugation (800 rpm) after 7–10 days and dissociated both mechanically and enzymatically (10 min in 0.05% trypsin, 0.53 mM EDTA-4Na).

### *In vivo* tumorigenic assay

The animal study protocol was reviewed and approved by the institutional animal care and use committee (IACUC) of the University of Pennsylvania. Six to eight week old female immune deficient mice were used in these studies. ALDH^low^ and ALDH^br^ (ALDH^bright^) cells were isolated by FACS sorting. Cells were suspended in phosphate buffered saline mixed with an equal volume of Matrigel (BD Biosciences) at 10 mg/ml. A total volume of 0.3 ml, containing 500; 5,000 or 50,000 cells, was injected subcutaneously (ovariectomized and oestrogen-pellet supplemented mice).

### MTT assay

MTT assays were performed in 96-well plates using the Cell Proliferation Kit (I) (Roche) following the manufacturer's instructions.

### Statistical analysis

Statistical analysis was performed using the SPSS and StatView statistical software packages.

## Results

### Expression and distribution of ALDH1 protein in normal human tissues

**Figure 1:**
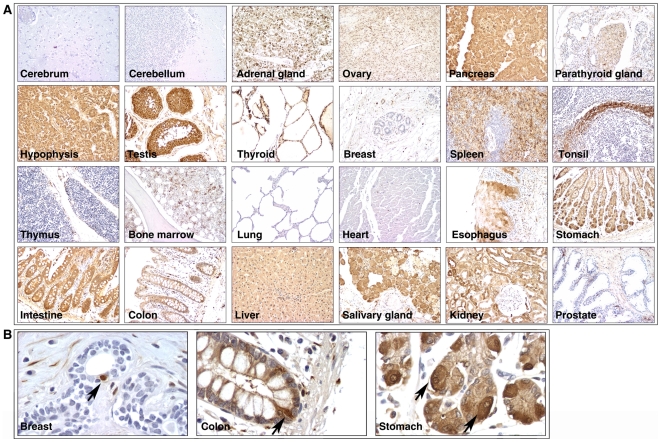
Expression and distribution of ALDH1 protein in normal human tissues. **A**. An FDA normal human organ tissue microarray was used to characterize the expression and distribution of ALDH1 in 24 tissues. **B**. Strongly positive ALDH1 cells were found in the areas in which epithelial stem/progenitor cells were putatively located in breast, colon and stomach.

As a stem cell marker, ALDH1 has been used to detect cancer stem cells in multiple types of human epithelial tumors using immunohistochemical staining; however, its expression and distribution patterns in normal human tissues are still largely unknown. To address this important question, we characterized the expression pattern of ALDH1 in normal human tissues by immunohistochemical staining. An FDA-approved normal human organ tissue microarray was used in this study. Here, 24 types of normal human organs were included based on FDA guidelines, where each organ was taken from 3 normal human individuals. Mouse anti-human ALDH1 antibody (clone: 44/ALDH) [34], [39], [46] was used to detect ALDH1 expression. As shown in Figure 1A, ALDH1 positive staining was detected mainly in the cytoplasm, and was widely expressed in the digestive system (including the epithelium of the esophagus, stomach, intestine and colon, as well as the liver and pancreas), the endocrine system (including the adrenal gland, hypophysis, thyroid and salivary gland) and the reproductive system (ovary and testis). There were no detectable ALDH1 positive cells in the cerebrum and cerebellum (Figure 1A). In other organs, ALDH1 was distributed in certain areas and in specific cell types. For example, in the spleen, ALDH1 positive cells were mainly found in leukocytes of the red pulp regions, but not in the white pulp (Figure 1A). Importantly, consistent with other reports [34], [46], [49], strongly ALDH1 positive cells were found in the areas in which epithelial stem/progenitor cells were putatively located in the breast, colon and stomach (Figure 1B). In normal breast, ALDH1 positive cells were mainly located in the luminal region (Figure 1B), and they were a relatively rare population (1–2% of luminal epithelial cells). Small ALDH1 positive cells were also found in normal breast stroma (Figure 1B). Double immunohistochemical staining demonstrated that most of these cells (>90%) were CD45 positive leukocytes (data not shown). In the normal colon, although weakly ALDH1 positive cells could be found in nearly all normal crypts, the number of strongly positive ALDH1 cells was quite limited (Figure 1B). These strongly positive cells were mainly located at the bases of the crypts (Figure 1B). Similar staining patterns are also seen in the stomach (Figure 1B).

### Expression and distribution of ALDH1 protein in human epithelial tumors

**Figure 2:**
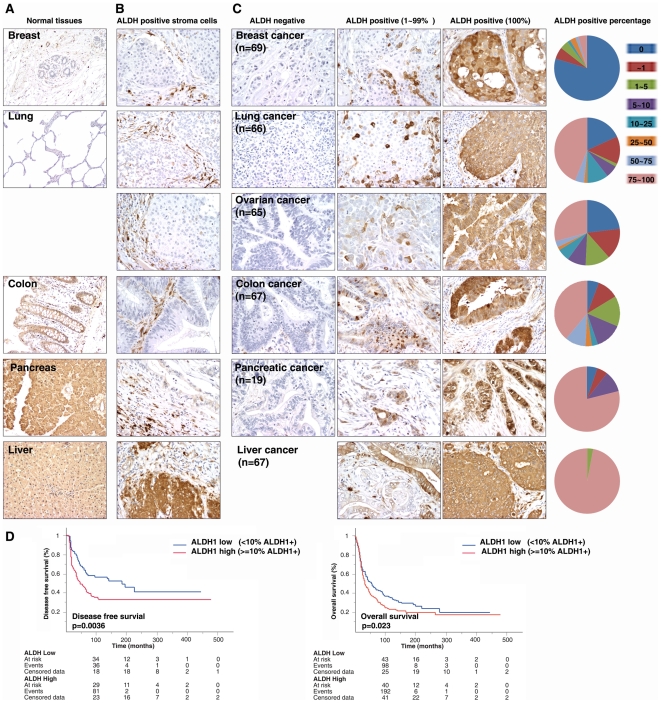
Expression and distribution of ALDH1 protein in human epithelial tumors. Tissue microarrays were used to characterize the expression and distribution of ALDH1 in cancers (n = 353). **A**. Expression of ALDH1 in the corresponding normal tissues. **B**. ALDH1+ tumor-infiltrating cells were detected in the stroma. **C**. Expression of ALDH1 in epithelial tumors. Percentage of ALDH1+ tumor cells was summarized. **D**. High percentage of ALDH1+ cells was associated with poor clinical outcomes in serous ovarian cancer.

Next, we investigated ALDH1 expression in human epithelial tumors using tissue arrays. As shown above, the patterns and levels of expression of ALDH1 in normal human tissues are remarkably different and can be classified into three types: 1) tissue with absent or limited ALDH1 expression, such as breast or lung; 2) tissue with relative weak ALDH1 expression, such as colon or gastric epithelium; and 3) tissue with extensive and high expression of ALDH1 such, as liver or pancreas (Figure 1A). Thus, we chose six types (breast, n = 69; lung, n = 66; ovarian, n = 65; colon, n = 67; liver, n = 67; and pancreatic cancer, n = 19) of epithelial cancers whose corresponding normal epitheliums were representative of each subtype. In all six tumor types, a large number of ALDH1 positive tumor-infiltrating stromal cells were found (Figure 2B). Double immunostaining demonstrated that most of these were CD45 positive cells. The percentage of ALDH1 positive tumor cells in tumor islets was independently quantified by two investigators with pathological training. Stromal ALDH1 positive cells were excluded from this analysis. We found that ALDH1 expression was negative in 79.7% (55/69) of breast, 18.2% (12/66) of lung and 23.1% (15/65) of ovarian cancers. On the contrary, only 6.0% (4/67) of colon and 5.3% (1/19) of pancreatic cancers were ALDH1 negative, and all liver cancer specimens were ALDH1 positive. In addition, for those ALDH positive tumors, the percentage of tumor cells expressing ALDH1 was also analyzed. The detailed percentage of ALDH1 positive cancer cells is summarized in Figure 2C. Except in the case of breast cancer (4.3%), a high percentage of ALDH1 expression (between 75 to 100% of tumor cells) was found in each of the other five cancer types (ovarian: 29.2%, colon: 38.8%, lung: 43.9%, pancreatic 78.9% and liver: 97.0%). Taken together, there was a clear correlation of ALDH1 expression between tumors and corresponding normal tissues. For example, in those cancer types where ALDH1 was highly expressed in the corresponding normal epitheliums, such as liver and pancreatic cancers, ALDH1 was expressed in most tumor specimens at remarkably high levels.

It has been reported by independent groups that a high percentage of ALDH1 positive tumor cells is associated with shorter survival times in breast [34], [37], [42], lung [38], pancreatic [40], bladder [41] and prostate [43] cancer patients. We tested the prognostic value of ALDH1 using a tissue array of serous ovarian cancers, the most common histotype of human epithelial ovarian cancer. The specimens were collected from the Department of Gynecology and Obstetrics at the Helsinki University Central Hospital. The median follow-up time of patients alive at the end of the study period was 101 months, ranging from 4 to 475 months. The patients were separated into two groups based on ALDH1 immunostaining: ALDH1 low (<10% tumor cells were ALDH1 positive) and ALDH1 high (≥10% tumor cells were ALDH1 positive). Consistent with the results from breast [34], [37], [42], lung [38], pancreatic [40], bladder [41] and prostate [43] cancer patients, we found that patients with high ALDH1 had shorter disease free and overall survival times compared to those with low ALDH1 (p = 0.0036 and p = 0.023, respectively, Figure 2D).

### ALDH1 enzymatic activity in epithelial tumor cells

**Figure 3:**
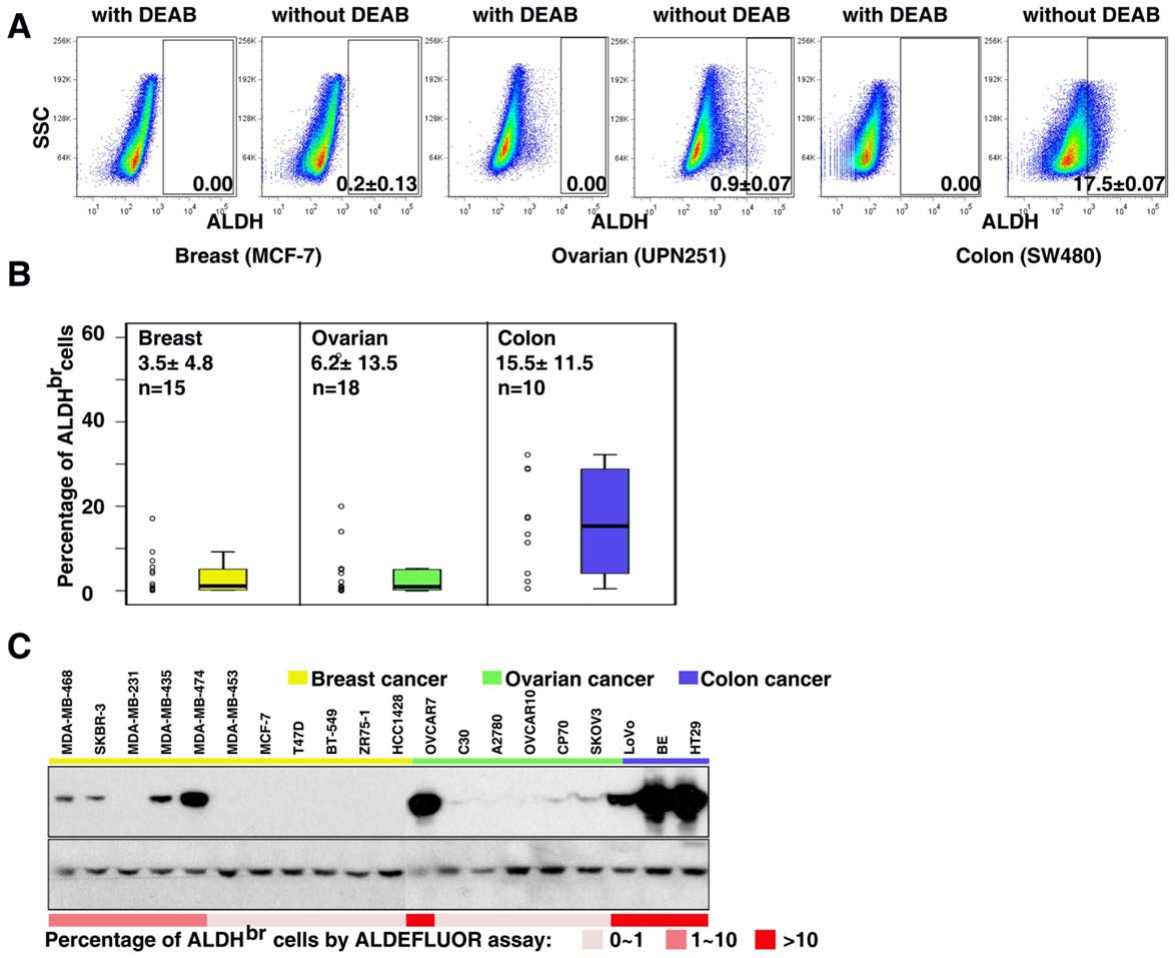
ALDH1 enzymatic activity and expression in established epithelial tumor cell lines. **A**. ALDH1 enzymatic activity was detected using the ALDEFLUOR assay. DEAB was used to inhibit the reaction of ALDH with the ALDEFLUOR reagent, providing a negative control. **B**. Summary of ALDH1 enzymatic activity in established breast (n = 15), ovarian (n = 18) and colon (n = 10) cancer cell lines. **C**. ALDH1 protein expression was detected by western blots. There was a positive correlation between ALDH1 protein expression and ALDH1 enzymatic activity. Full-length blots are presented in Supplemental Figure S3.

**Figure 4:**
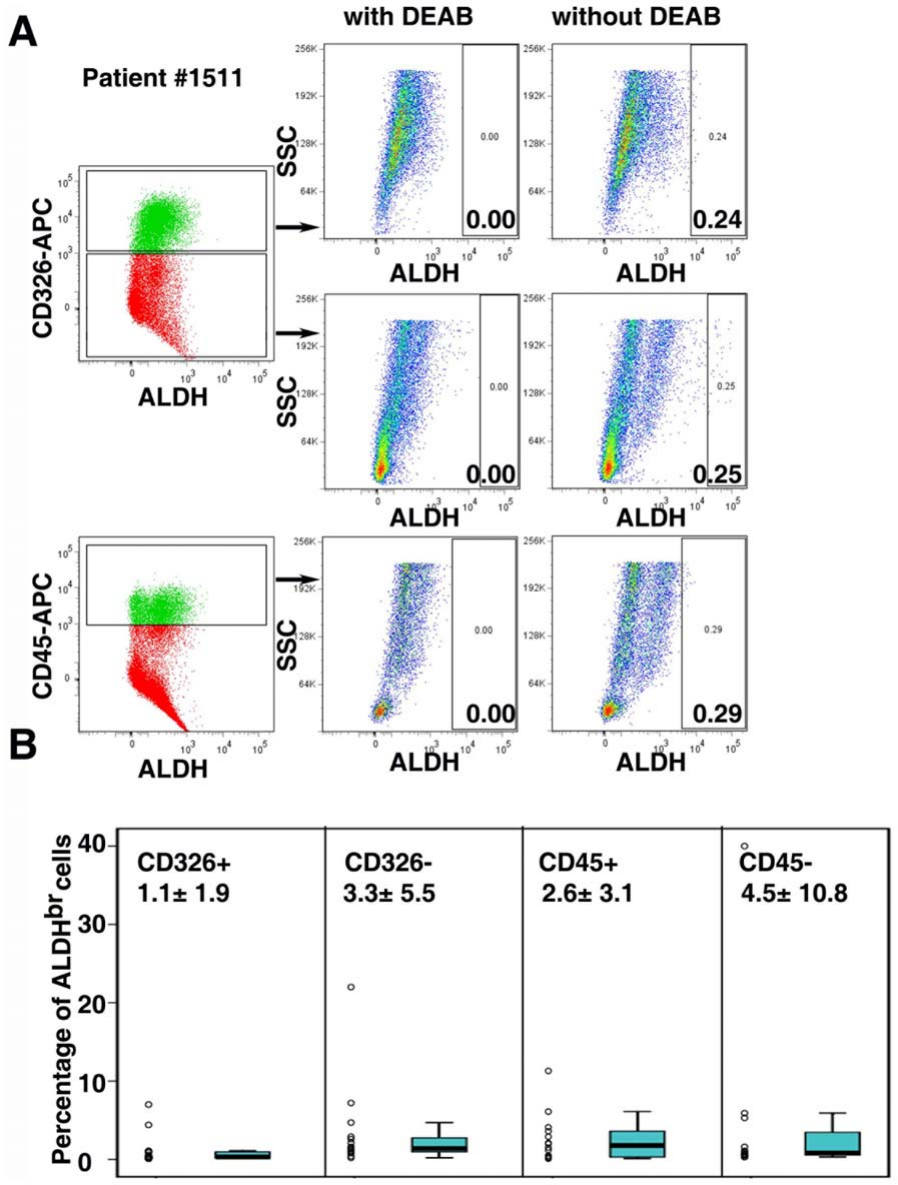
ALDH1 enzymatic activity in primary epithelial ovarian cancer cells. **A**. ALDH1 enzymatic activity in cells isolated from one ovarian cancer specimen. Dead cells were eliminated using magnetic beads and a MidiMACS separator. For immunophenotyping of ALDH^br^ ascites cells, APC-CD45 or APC-CD326 was used for counterstaining. DEAB was used to inhibit the reaction of ALDH with the ALDEFLUOR reagent, providing a negative control. **B**. Summary of the percentages of ALDH^br^ in each cell population from 16 late-stage ovarian cancer patients.

The ALDEFLUOR reagent, originally developed to detect ALDH activity in hematopoietic tissues [29], is known to act as a substrate for ALDH1. Recently, the ALDEFLUOR assay has been successfully applied to detect ALDH^br^ in cancer stem cells from non-hematopoietic tumors [34]. In the present study, we utilized this assay to examine ALDH1 enzymatic activity in epithelial tumor cells *in vitro*. First, to validate the results of the tissue array, we chose cell lines from three epithelial tumor types including breast (n = 15), ovarian (n = 18) and colon (n = 10). As shown in Figure 2, the percentage of tumors with a high frequency of ALDH1 positive cells was greater in colon cancer compared to breast or ovarian cancer. Consistent with this finding, we found that the percentage of ALDH^br^ cells in colon cancer cell lines (15.5±11.5%) was remarkably higher than in breast (3.5±4.8%) or ovarian (6.2±13.5%) cancer cell lines (Figure 3B and Table S1). To further confirm this result, we detected ALDH1 protein expression by western blots in these cell lines. As expected, ALDH1 protein expression levels were positively correlated with the percentage of ALDH^br^ cells in the cell lines (Figure 3C and Figure S3). Although there was a clear positive correlation between *in situ* immunostaining and the ALDEFLUOR assay results in these three tumor types, the percentage of cells exhibiting ALDH1 enzymatic activity by flow cytometry was smaller than the percentage of ALDH1 positive cells by immunostaining. For example, 4.3%, 29.2% and 38.8% of breast, ovarian and colon cancer specimens highly expressed ALDH1 (between 75 to 100% of tumor cells were ALDH1 positive, Figure 2C), however, none of the tumor cell lines were composed of greater than 75% of ALDH^br^ cells (Table S1). Next, we chose ovarian cancer as an example to investigate the ALDH enzymatic activity in primary tumor cells. Sixteen late stage ovarian cancer specimens were examined. Since we found that tumor-infiltrating leukocytes expressed high levels of ALDH1 (Figure 2B), we first separated ascites cells by CD326 (EpCAM, an epithelial marker to gate the tumor cell population) or CD45 (a lymphocyte maker to gate lymphocyte population) after removing dead cells using magnetic beads. The ALDEFLUOR assay was used to detect cells with ALDH activity in each of above population (Figure 4A). We found that 2.6±3.1% (0.1–11.3%) of CD45 positive cells were ALDH^br^ (Figure 4B). Consistent with the cell line study, 1.1±1.9% (0.1–7.0%) of CD326 positive tumor cells were ALDH^br^ (Figure 4B), a lower percentage than in the immunostaining results.

To address the question whether long-term *in vitro* culture will significantly affect ALDH activity in cancer cell lines, we chose an ovarian cancer transgenic mouse model, in which an oncogene (the transforming region of SV40) was overexpressed by the ovarian surface specific Müllerian inhibiting substance type II receptor (MISRII) promoter (Figure S1A). The ALDEFLUOR assay was performed on both primary isolated tumor cells and two long-term cultures of tumor cell lines (more than 40 passages) that were isolated from the same model. We found that there was no significant difference in the percentage of ALDH^br^ cells between primary tumors (9.5±7.6%, n = 5) and long-term cell cultures (6.8±4.9%, n = 2, P>0.05, Figure S1B). Since only limited numbers of cell lines were analyzed in the present study, this conclusion need further validate in other models.

Finally, we compared ALDH activity in ovarian cancer cells and their corresponding normal epitheliums, ovarian surface epithelial cells. Three immortalized human ovarian surface epithelial cell lines were compared to 18 human ovarian cancer cell lines, and primary isolated murine ovarian surface cells were compared to 7 murine ovarian cancer cell lines (including two lines from the MISIIR-SV40 transgenic mouse). We found that the percentage of ALDH^br^ cells was relatively higher in normal ovarian surface epithelial cells (human: 13.1±6.72%, n = 3; murine: 7.6%, n = 1) compared to ovarian cancer cells (human: 6.2±13.5%, n = 18; murine: 3.8±2.92%, Figure S2). Similarly, the percentage of ALDH^br^ cells was relatively higher in normal mammary epithelial cells (8.18±4.31%, n = 14) [34] compared to breast cancer cells (3.5±4.8%, n = 15, Figure 3).

### ALDH^br^ tumor cells have cancer stem cell properties and are resisted to chemotherapy

**Figure 5:**
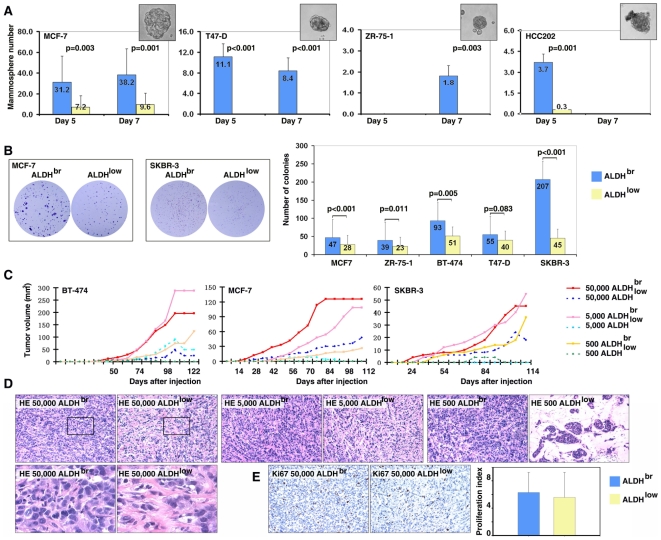
ALDH^br^ tumor cells exhibit cancer stem cell properties. **A**. ALDH^br^ cell populations (Blue) generated significantly higher numbers of mammospheres compared to the ALDH^low^ population (Yellow). **B**. Tumorigenicity of the ALDH^br^ and ALDH^low^ cells were examined *in vitro* using colony formation assays. **C**. Tumorigenicity of the ALDH^br^ and ALDH^low^ cells were examined *in vivo*. **D**. Histology of the ALDH^br^ and ALDH^low^ BT-474 tumors was examined by HE staining. **E**. Proliferation of the ALDH^br^ and ALDH^low^ BT-474 tumors was examined by Ki-67 immunostaining.

It has been demonstrated that ALDH^br^ tumor cells isolated from primary human tumors or from short-term passages of cells from NOD/SCID mice have cancer stem cell properties [34] and are resistant to chemotherapy [50], [51]. To examine the cancer stem cell properties of ALDH^br^ cells isolated from established long-term cultured cell lines, ALDH^low^ and ALDH^br^ tumor cells were isolated from established human breast and ovarian cancer cell lines by FACS sorting. First, their self-renewal ability was examined using the mammosphere assay [48] in four breast cancer cells. The number of mammospheres formed by ALDH^br^ tumor cells was significantly higher than by ALDH^low^ tumor cells (Figure 5A). ALDH^low^ cells from T47-D and ZR-75-1 cell lines lacked the ability to form mammospheres in suspension culture. Next, we examined their *in vitro* tumor growth capabilities using the colony formation assay in 5 breast cancer cell lines. ALDH^br^ tumor cells formed visibly larger colonies compared to ALDH^low^ tumor cells (Figure 5B). In addition, the colony numbers from ALDH^br^ tumor cells were significantly higher than from ALDH^low^ tumor cells (Figure 5B). Finally, the *in vivo* tumorigenic ability of the ALDH^low^ and ALDH^br^ tumor cells was examined using tumor cells that were graft-transplanted (500; 5,000; or 50,000) to immune deficient mice. We found that 500 ALDH^br^ but not ALDH^low^ tumor cells were capable of generating xenograft tumors *in vivo* (Figure 5C). Histology of three xenograft tumors was examined by HE staining. In MCF-7 and SKBR-3 cell lines, there was no significant histological difference between ALDH^br^ and ALDH^low^ tumors. However, in BT-474 tumors, there were limited stromal cells in ALDH^br^ tumors compared to ALDH^low^ tumors (Figure 5D). *In vivo* cell proliferation was also examined using Ki-67 staining. We did not find any significant difference in the proliferation index (percentage of Ki-67 positive cells) between the ALDH^br^ and ALDH^low^ tumors in all three cell lines (Figure 5E).

**Figure 6:**
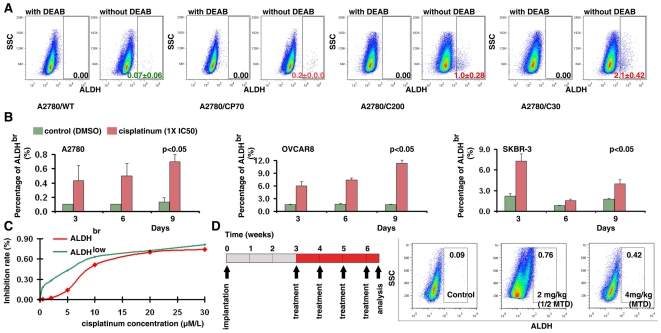
ALDH^br^ tumor cells are resistant to the chemotherapeutic drug platinum. **A**. ALDH^br^ cells were remarkably expanded in platinum resistant cell lines (A2780/CP70, A2780/C200 and A2780/C30) compared to their parental platinum sensitive line (A2780/WT). **B**. *In vitro* platinum treatment significantly increased the ALDH^br^ cell population in ovarian and breast cancer cell lines after three days. **C**. ALDH^br^ cells were resistant to platinum treatment. ALDH^br^ and ALDH^low^ cells were isolated by FACS sorting. **D**. ALDH^br^ tumor cell population was resistant to chemotherapy *in vivo*. The q7d×4 i.p. treatment schedule was used for the experimental therapy. Tumors were collected and disassociated by enzyme digestion. Percentage of the ALDH^br^ population was analyzed by ALDEFLUOR assay.

Rapidly accumulating evidence suggests that cancer stem cells may be highly resistant to radiation or chemotherapy [18], [19], [20]. Consistent with these findings, we found that the ALDH^br^ cell population is expanded in a set of platinum resistant ovarian cancer cell lines, A2780/CP70, A2780/C200 and A2780/C30, compared to their parental platinum sensitive line, A2780/WT (Figure 6A). Furthermore, treatment of cells with cisplatinum (1XIC_50_) significantly enriched the ALDH^br^ cell population in ovarian and breast cancer cell lines *in vitro* (Figure 6B). The ALDH^br^ population was significantly more resistant to platinum treatment compared to the ALDH^low^ population (Figure 6C). Lastly, we test the above observation *in vivo* using an ovarian cancer xenograft mouse model. Three weeks after the implantation of tumor cells, A2780 (5×10^6^ per animal), the mice were randomly assigned to three experimental groups to receive treatment by i.p. injection with 1) control treatment (n = 4), 2) 2 mg/kg cis-platinum treatment (½ maximum tolerated dose (MTD), n = 3) and 3) 4 mg/kg cis-platinum treatment (full MTD, n = 3). The q7d×4 i.p. treatment schedule (Figure 6D) was used for the experimental therapy. Percentage of the ALDH positive population was analyzed by ALDEFLUOR assay. Consistent with our *in vitro* data, cis-platinum treatment significantly increased the ALDH^br^ cell population in xenograft tumor *in vitro* (Figure 6D, ½ MTD: 8.44-fold in average; full MTD: 4.67-fold in average, compared to non-treated tumors). It suggests that ALDH^br^ tumor cell population is resistant to chemotherapy. Similar results was also reported in colon cancer *in vivo* most recently by an independent group [50].

## Discussion

As a novel approach, the ALDEFLUOR assay and ALDH1 immunostaining may prove useful for the detection and isolation of cancer stem cells in epithelial tumors, thus facilitating the application of cancer stem cell concepts to clinical practice [34]. We found that pattern and level of expression of ALDH1 in normal human tissues are remarkably different and could be classified into three types: 1) tissue with absent or limited ALDH1 expression, such as breast or lung; 2) tissue with relative weak ALDH1 expression, such as colon or gastric epitheliums; and 3) tissue with extensive and high expression of ALDH1, such as liver or pancreas (Figure 1A). In agreement with other reports [34], [46], [49], we found that strongly positive ALDH1 cells can be clearly identified in regions where epithelial stem/progenitor cells are putatively located in the first two types of tissues (Figure 1B). Ginestier *et al.* has demonstrated that these ALDH1 positive cells are indeed epithelial progenitor cells [34]. Taken together, ALDH1 can serve as a marker to detect stem/progenitor cells *in situ* in those tissues where ALDH1 expression is limited (such as breast) or weak (such as colon). However, for the tissues with wide and strong expression of ALDH1 (such as liver and pancreas), ALDH1 may not be able to be serve as a stem cell marker.

Importantly, we found that the percentage of ALDH1 positive tumor cells was significantly correlated with the level of ALDH1 expression in corresponding normal tissues. For example, ALDH1 is expressed in limited cell populations of the breast, lung or ovary. In each of these cancer types, the percentage of ALDH1 negative tumor cells was significantly larger than in colon cancer, whose corresponding tissue, colon epithelium, expressed relatively higher ALDH1 (Figure 2). Furthermore, in other two types of cancers examined in our study, liver and pancreas, almost all tumor cells were ALDH1 positive, while the corresponding normal tissues expressed extensive, high levels of ALDH1 (Figure 2). This finding was further confirmed *in vitro* by the ALDEFUOR assay. Tumor cells derived from tumors exhibiting high ALDH1 levels contained a significantly higher frequency of ALDH^br^ cells compared to tumors expression low ALDH1 levels (Figure 3 A and B). These results indicate that as a cancer stem cell marker, ALDH1 may be only suitable for those tumors whose corresponding normal tissues express a low background level of ALDH1. Finally, increasing evidence indicates that higher percentage of ALDH1 positive tumor cells may serve as a novel prognostic marker associated with poor clinical outcome in multiple human solid cancers including breast [34], [37], [42], lung [38], pancreatic [40], bladder [41] and prostate [43] cancers. Meanwhile, Chang *et al.* have reported the ALDH1 expression correlates with favorable prognosis in ovarian cancer including 266 serous ovarian cancer patients and 176 non-serous ovarian cancer patients [39]. Consistent with the reports in multiple types of tumors [34], [37], [38], [40], [41], [42], [43], we found that higher percentage of ALDH1 positive tumor cells is significantly associated with poor clinical outcome in serous ovarian cancer (Figure 2D, n = 439, p = 0.0036). It indicates that the prognostic value of ALDH1 in ovarian cancer, *albeit potent*, may well be histotype-type specific. Based on clinicopathological and molecular studies, different histotype ovarian epithelial tumors have remarkably distinct molecular background and biological behavior. They may be considered as different diseases, although they are located in the same anatomic location. Therefore, the prognostic value of ALDH1 in different types of ovarian cancers is still needed to be further investigated in large-scale independent sample sets.

Using the ALDEFLUOR assay, we examined ALDH1 enzymatic activity in both established and primary cancer cell lines. We found that ALDH1 enzymatic activity was positively correlated with ALDH1 expression in cancer cells (Figure 3C). Although we found that ALDH1 activity was relatively higher in the established ovarian cancer cell lines compared to primary ovarian tumor specimens (Figure 3B and Figure 4B), we did not find a significant difference in ALDH1 activity between primary isolated tumors and long-term cultures of tumor cell lines from the same ovarian cancer transgenic mouse model (Figure S1), suggesting that the relatively lower ALDH enzymatic activity observed in primary ovarian cancer cells may be due to the procedure of sample collection and preparation. Interestingly, we found that the ALDH1 enzymatic activity was relatively lower in ovarian cancer cell lines compared to normal ovarian surface epithelial cells (Figure S2). Similar results were also found in breast cancer [34].

It has been demonstrated that ALDH^br^ tumor cells isolated from primary tumors have cancer stem cell properties such as self-renewal and tumorigenicity [34]. In the present study, we examined these properties in the ALDH^br^ cells isolated from established tumor cell lines. Similar to the results based on primary tumors [34], ALDH^br^ tumor cells from breast cancer cell lines generated a significantly higher number of tumorspheres *in vitro*, and were more tumorigenic *in vitro* and *in vivo* (Figure 5). In agreement with our findings, several groups also reported that ALDH^br^ cell populations isolated from established cancer cell lines had more tumorigenic and metastatic capacities [42], [52], [53] Finally, we demonstrated that ALDH^br^ ovarian cancer cells were resistant to chemotherapeutic drugs such as platinum (Figure 6). Both long-term and short-term platinum treatments led to an enrichment of the ALDH^br^ tumor cell population. This result is consistent with findings in other cancers [50], [51], and indicates that the development of more effective therapies for cancer requires effective targeting of this cell population.

## Supporting Information

Table S1 Summary of percentage of ALDH bright cells in cell lines (n = 54).
(0.09 MB DOC)

Figure S1 ALDH1 enzymatic activity in murine primary ovarian cancer cells and long-term cultured cell lines from a transgenic animal. A. An ovarian cancer transgenic mouse model expressing the transforming region of SV40 under control of the MISRII promoter was used in this study. Bilateral ovarian tumors developed at 15∼17 weeks post birth. B. Summary of the percentage of ALDHbr cells in primary ovarian cancer cells and long-term cultured cell lines from same tumor model.
(0.22 MB TIF)

Figure S2 ALDH1 enzymatic activity in ovarian surface epithelial cells and epithelial ovarian caner cell lines. Summary of the percentage of ALDHbr cells in human (left) and murine (right) ovarian surface epithelial cells and epithelial ovarian caner cell lines.
(0.17 MB TIF)

Figure S3 Full-length blots of figure 3C.
(0.86 MB TIF)

## References

[1] Lapidot T, Sirard C, Vormoor J, Murdoch B, Hoang T (1994). A cell initiating human acute myeloid leukaemia after transplantation into SCID mice.. Nature.

[2] Bonnet D, Dick JE (1997). Human acute myeloid leukemia is organized as a hierarchy that originates from a primitive hematopoietic cell.. Nat Med.

[3] Al-Hajj M, Wicha MS, Benito-Hernandez A, Morrison SJ, Clarke MF (2003). Prospective identification of tumorigenic breast cancer cells.. Proc Natl Acad Sci U S A.

[4] Singh SK, Hawkins C, Clarke ID, Squire JA, Bayani J (2004). Identification of human brain tumour initiating cells.. Nature.

[5] Fang D, Nguyen TK, Leishear K, Finko R, Kulp AN (2005). A tumorigenic subpopulation with stem cell properties in melanomas.. Cancer Res.

[6] Szotek PP, Pieretti-Vanmarcke R, Masiakos PT, Dinulescu DM, Connolly D (2006). Ovarian cancer side population defines cells with stem cell-like characteristics and Mullerian Inhibiting Substance responsiveness.. Proc Natl Acad Sci U S A.

[7] Dalerba P, Dylla SJ, Park IK, Liu R, Wang X (2007). Phenotypic characterization of human colorectal cancer stem cells.. Proc Natl Acad Sci U S A.

[8] O'Brien CA, Pollett A, Gallinger S, Dick JE (2007). A human colon cancer cell capable of initiating tumour growth in immunodeficient mice.. Nature.

[9] Ricci-Vitiani L, Lombardi DG, Pilozzi E, Biffoni M, Todaro M (2007). Identification and expansion of human colon-cancer-initiating cells.. Nature.

[10] Szotek PP, Chang HL, Brennand K, Fujino A, Pieretti-Vanmarcke R (2008). Normal ovarian surface epithelial label-retaining cells exhibit stem/progenitor cell characteristics.. Proc Natl Acad Sci U S A.

[11] Smalley M, Ashworth A (2003). Stem cells and breast cancer: A field in transit.. Nat Rev Cancer.

[12] Jordan CT, Guzman ML, Noble M (2006). Cancer stem cells.. N Engl J Med.

[13] Lobo NA, Shimono Y, Qian D, Clarke MF (2007). The biology of cancer stem cells.. Annu Rev Cell Dev Biol.

[14] Stingl J, Caldas C (2007). Molecular heterogeneity of breast carcinomas and the cancer stem cell hypothesis.. Nat Rev Cancer.

[15] Ward RJ, Dirks PB (2007). Cancer stem cells: at the headwaters of tumor development.. Annu Rev Pathol.

[16] Visvader JE, Lindeman GJ (2008). Cancer stem cells in solid tumours: accumulating evidence and unresolved questions.. Nat Rev Cancer.

[17] Lee JT, Herlyn M (2007). Old disease, new culprit: tumor stem cells in cancer.. J Cell Physiol.

[18] Bao S, Wu Q, McLendon RE, Hao Y, Shi Q (2006). Glioma stem cells promote radioresistance by preferential activation of the DNA damage response.. Nature.

[19] Li X, Lewis MT, Huang J, Gutierrez C, Osborne CK (2008). Intrinsic resistance of tumorigenic breast cancer cells to chemotherapy.. J Natl Cancer Inst.

[20] Diehn M, Cho RW, Lobo NA, Kalisky T, Dorie MJ (2009). Association of reactive oxygen species levels and radioresistance in cancer stem cells.. Nature.

[21] Dontu G (2008). Breast cancer stem cell markers - the rocky road to clinical applications.. Breast Cancer Res.

[22] Schatton T, Murphy GF, Frank NY, Yamaura K, Waaga-Gasser AM (2008). Identification of cells initiating human melanomas.. Nature.

[23] Yang ZF, Ho DW, Ng MN, Lau CK, Yu WC (2008). Significance of CD90+ cancer stem cells in human liver cancer.. Cancer Cell.

[24] Vaillant F, Asselin-Labat ML, Shackleton M, Forrest NC, Lindeman GJ (2008). The mammary progenitor marker CD61/beta3 integrin identifies cancer stem cells in mouse models of mammary tumorigenesis.. Cancer Res.

[25] Vlashi E, Kim K, Lagadec C, Donna LD, McDonald JT (2009). In vivo imaging, tracking, and targeting of cancer stem cells.. J Natl Cancer Inst.

[26] Goodell MA, Brose K, Paradis G, Conner AS, Mulligan RC (1996). Isolation and functional properties of murine hematopoietic stem cells that are replicating in vivo.. J Exp Med.

[27] Hadnagy A, Gaboury L, Beaulieu R, Balicki D (2006). SP analysis may be used to identify cancer stem cell populations.. Exp Cell Res.

[28] Yoshida A, Rzhetsky A, Hsu LC, Chang C (1998). Human aldehyde dehydrogenase gene family.. Eur J Biochem.

[29] Storms RW, Trujillo AP, Springer JB, Shah L, Colvin OM (1999). Isolation of primitive human hematopoietic progenitors on the basis of aldehyde dehydrogenase activity.. Proc Natl Acad Sci U S A.

[30] Hess DA, Meyerrose TE, Wirthlin L, Craft TP, Herrbrich PE (2004). Functional characterization of highly purified human hematopoietic repopulating cells isolated according to aldehyde dehydrogenase activity.. Blood.

[31] Armstrong L, Stojkovic M, Dimmick I, Ahmad S, Stojkovic P (2004). Phenotypic characterization of murine primitive hematopoietic progenitor cells isolated on basis of aldehyde dehydrogenase activity.. Stem Cells.

[32] Gentry T, Foster S, Winstead L, Deibert E, Fiordalisi M (2007). Simultaneous isolation of human BM hematopoietic, endothelial and mesenchymal progenitor cells by flow sorting based on aldehyde dehydrogenase activity: implications for cell therapy.. Cytotherapy.

[33] Corti S, Locatelli F, Papadimitriou D, Donadoni C, Salani S (2006). Identification of a primitive brain-derived neural stem cell population based on aldehyde dehydrogenase activity.. Stem Cells.

[34] Ginestier C, Hur MH, Charafe-Jauffret E, Monville F, Dutcher J (2007). ALDH1 Is a Marker of Normal and Malignant Human Mammary Stem Cells and a Predictor of Poor Clinical Outcome.. Cell Stem Cell.

[35] Ibarra I, Erlich Y, Muthuswamy SK, Sachidanandam R, Hannon GJ (2007). A role for microRNAs in maintenance of mouse mammary epithelial progenitor cells.. Genes Dev.

[36] Burger PE, Gupta R, Xiong X, Ontiveros CS, Salm SN (2009). High ALDH Activity: A Novel Functional Marker of Murine Prostate Stem/Progenitor Cells.. Stem Cells.

[37] Morimoto K, Kim SJ, Tanei T, Shimazu K, Tanji Y (2009). Stem cell marker aldehyde dehydrogenase 1-positive breast cancers are characterized by negative estrogen receptor, positive human epidermal growth factor receptor type 2, and high Ki67 expression.. Cancer Sci.

[38] Jiang F, Qiu Q, Khanna A, Todd NW, Deepak J (2009). Aldehyde dehydrogenase 1 is a tumor stem cell-associated marker in lung cancer.. Mol Cancer Res.

[39] Chang B, Liu G, Xue F, Rosen DG, Xiao L (2009). ALDH1 expression correlates with favorable prognosis in ovarian cancers.. Mod Pathol.

[40] Rasheed ZA, Yang J, Wang Q, Kowalski J, Freed I (2010). Prognostic significance of tumorigenic cells with mesenchymal features in pancreatic adenocarcinoma.. J Natl Cancer Inst.

[41] Su Y, Qiu Q, Zhang X, Jiang Z, Leng Q (2010). Aldehyde dehydrogenase 1 A1-positive cell population is enriched in tumor-initiating cells and associated with progression of bladder cancer.. Cancer Epidemiol Biomarkers Prev.

[42] Charafe-Jauffret E, Ginestier C, Iovino F, Tarpin C, Diebel M (2010). Aldehyde dehydrogenase 1-positive cancer stem cells mediate metastasis and poor clinical outcome in inflammatory breast cancer.. Clin Cancer Res.

[43] Li T, Su Y, Mei Y, Leng Q, Leng B (2010). ALDH1A1 is a marker for malignant prostate stem cells and predictor of prostate cancer patients' outcome.. Lab Invest.

[44] Roby KF, Taylor CC, Sweetwood JP, Cheng Y, Pace JL (2000). Development of a syngeneic mouse model for events related to ovarian cancer.. Carcinogenesis.

[45] Connolly DC, Bao R, Nikitin AY, Stephens KC, Poole TW (2003). Female mice chimeric for expression of the simian virus 40 TAg under control of the MISIIR promoter develop epithelial ovarian cancer.. Cancer Res.

[46] Huang EH, Hynes MJ, Zhang T, Ginestier C, Dontu G (2009). Aldehyde dehydrogenase 1 is a marker for normal and malignant human colonic stem cells (SC) and tracks SC overpopulation during colon tumorigenesis.. Cancer Res.

[47] Zhang L, Yang N, Garcia JR, Mohamed A, Benencia F (2002). Generation of a syngeneic mouse model to study the effects of vascular endothelial growth factor in ovarian carcinoma.. Am J Pathol.

[48] Dontu G, Abdallah WM, Foley JM, Jackson KW, Clarke MF (2003). In vitro propagation and transcriptional profiling of human mammary stem/progenitor cells.. Genes Dev.

[49] Carpentino JE, Hynes MJ, Appelman HD, Zheng T, Steindler DA (2009). Aldehyde dehydrogenase-expressing colon stem cells contribute to tumorigenesis in the transition from colitis to cancer.. Cancer Res.

[50] Dylla SJ, Beviglia L, Park IK, Chartier C, Raval J (2008). Colorectal cancer stem cells are enriched in xenogeneic tumors following chemotherapy.. PLoS ONE.

[51] Tanei T, Morimoto K, Shimazu K, Kim SJ, Tanji Y (2009). Association of breast cancer stem cells identified by aldehyde dehydrogenase 1 expression with resistance to sequential Paclitaxel and epirubicin-based chemotherapy for breast cancers.. Clin Cancer Res.

[52] Charafe-Jauffret E, Ginestier C, Iovino F, Wicinski J, Cervera N (2009). Breast cancer cell lines contain functional cancer stem cells with metastatic capacity and a distinct molecular signature.. Cancer Res.

[53] Wang L, Park P, Zhang H, La Marca F, Lin CY (2010). Prospective identification of tumorigenic osteosarcoma cancer stem cells in OS99-1 cells based on high aldehyde dehydrogenase activity.. Int J Cancer.

