# Supplementary material for: Distinct Expression Levels and Patterns of Stem Cell Marker, Aldehyde Dehydrogenase Isoform 1 (ALDH1), in Human Epithelial Cancers
Source: PLoS One. 2010 Apr 21;5(4):e10277. doi: 10.1371/journal.pone.0010277 (PMC2858084; doi:10.1371/journal.pone.0010277)
Supplement: Table S1 — Summary of percentage of ALDH bright cells in cell lines (n = 54). (0.09 MB DOC) [file pone.0010277.s001.doc]

**Table S1. Summary of percentage of ALDH^br^** cells in cell lines (n=54).

| **Cell Type** | **Cell Line Name** | **Percentage of ALDH bright cells** | |
| --- | --- | --- | --- |
|  |  | **Mean** | **SD** |
| **Human breast cancer** | BT-20 | 5.60 | 0.92 |
| **(n=15)** | BT-474 | 17.10 | 8.30 |
|  | BT-483 | 9.20 | 5.58 |
|  | BT-549 | 0.10 | 0.15 |
|  | HCC1428 | 0.10 | 0.00 |
|  | HCC202 | 0.70 | 0.35 |
|  | MCF-7 | 0.20 | 0.13 |
|  | MDA-MB-157 | 4.60 | 1.48 |
|  | MDA-MB-231 | 1.10 | 0.06 |
|  | MDA-MB-436 | 1.60 | 0.85 |
|  | MDA-MB-453 | 0.10 | 0.07 |
|  | MDA-MB-468 | 4.20 | 3.76 |
|  | SKBR-3 | 7.10 | 1.20 |
|  | T-47D | 0.40 | 0.15 |
|  | ZR-75-1 | 0.20 | 0.14 |
| **Human ovarian cancer** | 2008 | 0.22 | 0.16 |
| **(n=18)** | A1847 | 0.01 | 0.00 |
|  | A2780 | 0.07 | 0.06 |
|  | A2780/C200 | 1.00 | 0.28 |
|  | A2780/C30 | 2.10 | 0.42 |
|  | A2780/CP70 | 0.20 | 0.00 |
|  | OVCAR2 | 4.15 | 1.45 |
|  | OVCAR3 | 5.25 | 0.07 |
|  | OVCAR4 | 0.60 | 0.00 |
|  | OVCAR5 | 0.90 | 0.07 |
|  | OVCAR7 | 55.75 | 12.23 |
|  | OVCAR8 | 5.00 | 0.07 |
|  | OVCAR10 | 0.20 | 0.07 |
|  | OWA42 | 0.20 | 0.07 |
|  | PE01 | 20.00 | 4.18 |
|  | PE04 | 14.00 | 3.44 |
|  | SKOV3 | 0.30 | 0.00 |
|  | UPN251 | 0.90 | 0.07 |
| **Human colon cancer** | BE | 28.80 | 11.95 |
| **(n=10)** | DLD1 | 0.50 | 0.07 |
|  | HCT15 | 2.20 | 0.70 |
|  | HT29 | 29.00 | 14.32 |
|  | HTC116 | 13.40 | 6.07 |
|  | LoVo | 17.20 | 5.30 |
|  | SW1222 | 4.10 | 0.78 |
|  | SW480 | 17.50 | 0.07 |
|  | SW620 | 11.40 | 2.90 |
|  | WiDr | 32.20 | 4.53 |
| **Human ovarian epithelium** | IOSE7576 | 6.03 | 3.27 |
| **(n=3)** | IOSE386 | 13.73 | 4.02 |
|  | IOSE398 | 19.43 | 3.00 |
| **Mouse ovarian epithelium** | MOSE | 7.60 | 3.77 |
| **Mouse ovarian cancer** | MOVCAR5009 | 3.30 | 0.31 |
| **(n=7)** | MOVCAR12-3 | 10.20 | 3.11 |
|  | C1 | 1.60 | 0.07 |
|  | C1-AKT | 2.60 | 0.90 |
|  | C2 | 2.37 | 0.50 |
|  | C2-Her2/neo | 4.10 | 0.72 |
|  | ID8 | 2.60 | 0.61 |
