# Supplementary figures and images for: Distinct Expression Levels and Patterns of Stem Cell Marker, Aldehyde Dehydrogenase Isoform 1 (ALDH1), in Human Epithelial Cancers
Source: PLoS One. 2010 Apr 21;5(4):e10277. doi: 10.1371/journal.pone.0010277 (PMC2858084; doi:10.1371/journal.pone.0010277)

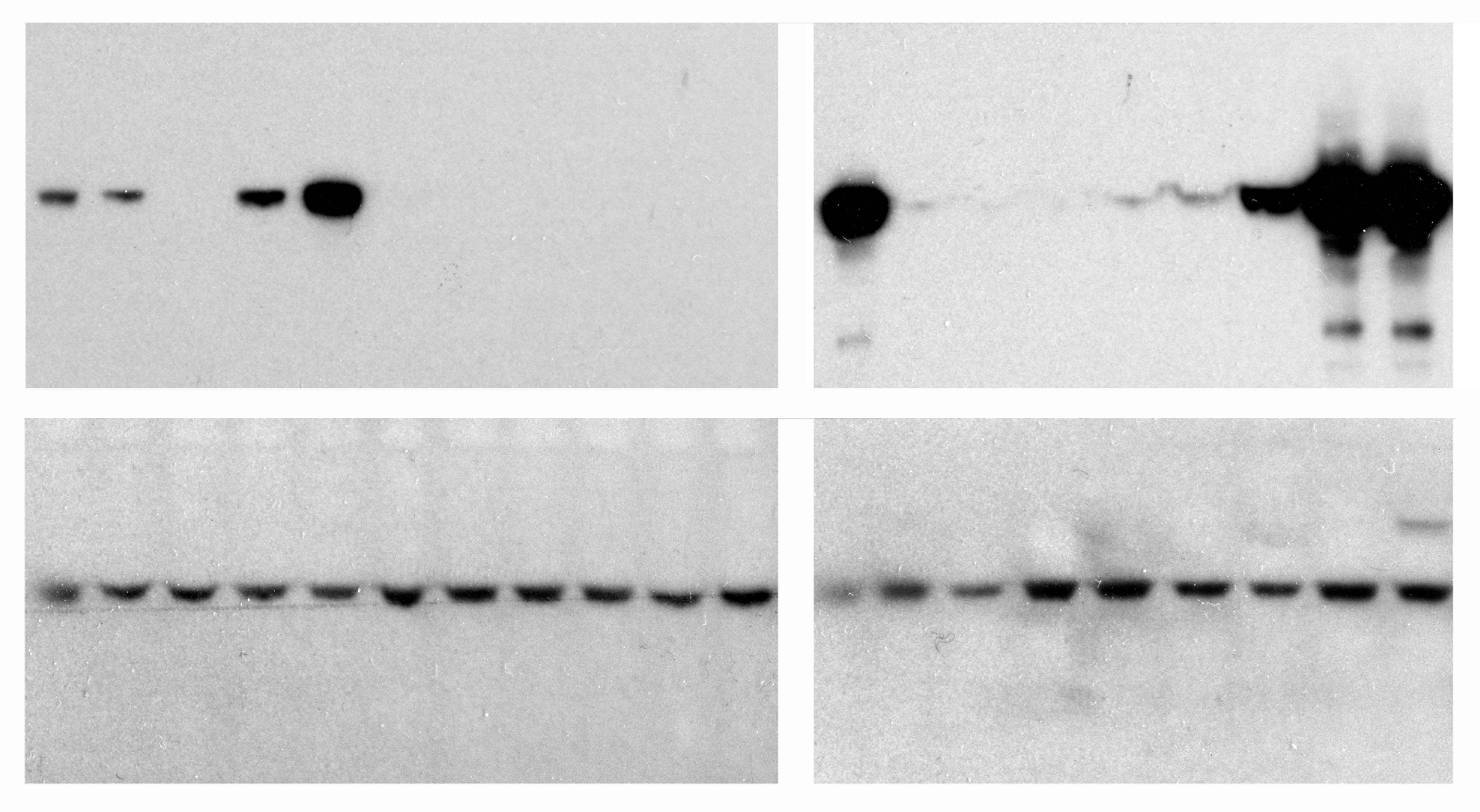

Supplement: Figure S3 — Full-length blots of figure 3C. (0.86 MB TIF) [file pone.0010277.s004.tif]
